# An Optimized Liquid Chromatography–Mass Spectrometry Method for Ganglioside Analysis in Cell Lines

**DOI:** 10.3390/cells13191640

**Published:** 2024-10-02

**Authors:** Akeem Sanni, Andrew I. Bennett, Yifan Huang, Isabella Gidi, Moyinoluwa Adeniyi, Judith Nwaiwu, Min H. Kang, Michelle E. Keyel, ChongFeng Gao, C. Patrick Reynolds, Brian Haab, Yehia Mechref

**Affiliations:** 1 Chemistry and Biochemistry Department, Texas Tech University, Lubbock, TX 79409, USA; aksanni@ttu.edu (A.S.); andy.bennett@ttu.edu (A.I.B.); yifan.huang@ttu.edu (Y.H.); isabellagidi@college.havard.edu (I.G.); jnwaiwu@ttu.edu (J.N.); 2 Cancer Center, School of Medicine, Texas Tech University Health Sciences Center (TTUHSC), Lubbock, TX 79416, USA; min.kang@ttuhsc.edu (M.H.K.); patrick.reynolds@ttuhsc.edu (C.P.R.); 3 Van Andel Institute, Grand Rapids, MI 49503, USA; chongfeng.gao@vai.org (C.G.); brian.haab@vai.org (B.H.)

**Keywords:** gangliosides, ZIC-HILIC, LC-MS, neuroblastoma

## Abstract

Gangliosides are glycosphingolipids composed of a sialylated glycan head group and a ceramide backbone. These anionic lipids form lipid rafts and play crucial roles in regulating various proteins involved in signal transduction, adhesion, and cell–cell recognition. Neuroblastoma, a pediatric cancer of the sympathetic nervous system, is treated with intensive chemotherapy, radiation, and an antibody targeting the GD2 ganglioside. Gangliosides are critical in neuroblastoma development and serve as therapeutic targets, making it essential to establish a reliable, rapid, and cost-effective method for profiling gangliosides, particularly one capable of isomeric separation of intact species. In this study, liquid chromatography–mass spectrometry (LC-MS) was optimized using standard gangliosides, followed by the optimization of sphingolipid extraction methods from cell lines by comparing Folch and absolute methanol extraction techniques. Percent recovery and the number of identified sphingolipids were used to evaluate the analytical merits of these methods. A standard gangliosides calibration curve demonstrated excellent linearity (R^2^ = 0.9961–0.9975). The ZIC-HILIC column provided the best separation of ganglioside GD1 isomers with a 25 min runtime. GD1a elutes before GD1b on the ZIC-HILIC column. Absolute methanol yielded better percent recovery (96 ± 7) and identified 121 different sphingolipids, the highest number between the two extraction methods. The optimized method was applied to profile gangliosides in neuroblastoma (COG-N-683), pancreatic cancer (PSN1), breast cancer (MDA-MB-231BR), and brain tumor (CRL-1620) cell lines. The ganglioside profile of the neuroblastoma cell line COG-N-683 showed an inverse relationship between GD1 and GD2. Ceramide, Hex1Cer, GM1, and GM3 were highly abundant in CRL-1620, PSN1, and MDA-MB-231BR, respectively. These results suggest that our method provides a sensitive, reliable, and high-throughput workflow for ganglioside profiling across different cell types.

## 1. Introduction

The glycobiology of the Central Nervous System (CNS) is primarily composed of glycolipids, including gangliosides and other sphingolipids, in contrast to the peripheral systems where glycoproteins dominate [1,2]. Gangliosides are a type of glycosphingolipid consisting of a glycan headgroup with sialic acid residues attached via glycosidic linkage and a hydrophobic ceramide tail that anchors them to the cell membrane. This unique structure allows gangliosides to interact with proteins and other glycans on the same membrane (*cis* interactions) and with molecules on neighboring cells, thereby regulating cell signaling and communication [3,4]. The gangliosides GM1, GD1a, GD1b, and GT1b are predominant in the vertebrate brain [5].

Changes in ganglioside levels have been implicated in aging [6,7] and neurodegenerative diseases [8], including Parkinson’s disease (PD) [9,10], Alzheimer’s disease (AD) [11,12,13], and Huntington’s disease (HD) [14,15]. GM1, a ganglioside abundant in the brain, has shown neuroprotective effects when administered in models of neuronal injury and neurodegeneration [8,12,16].

Despite their importance, gangliosides are less studied than other biomolecules, such as nucleic acids and proteins, primarily due to experimental challenges. Key challenges in the field include the need for less expensive and more efficient methods for recovering gangliosides from complex biological samples [5] and the need for improved accuracy and precision in analyzing specific ganglioside entities. These challenges arise from the low concentration of gangliosides in some samples compared to other biomolecules and the presence of a complex matrix of biomolecules that can interfere with the efficiency of ganglioside extraction and analysis, creating background noise that hinders detection [13].

Traditionally, ganglioside profiles have been studied using thin-layer chromatography followed by densitometric quantitation [17,18]. However, this technique has several limitations, including limited resolution, sensitivity, and lack of structural information, all of which are crucial for comprehensive and quantitative analysis of gangliosides. Liquid chromatography–mass spectrometry (LC-MS) has gained significant attention in “omics” fields, including “gangliosidomics”. LC-MS combines the high separation power of liquid chromatography with the sensitivity and accuracy of mass spectrometry to separate, detect, and identify a wide range of compounds in a sample [13,19]. It allows for high-throughput analysis, structural characterization, and quantification of gangliosides. By employing various chromatographic columns and mobile phase compositions, efficient separation of gangliosides and their isomers is feasible, with reduced interference from other lipid species or biomolecules in the biological sample [5]. Despite the power of these methods, challenges remain, particularly in the separation of ganglioside isomers. Gangliosides can have varying *m*/*z* values, and sample preparation may require antibody tagging, adding complexity to the analysis.

In this work, we developed and optimized methods to address these challenges, focusing on efficient, low-cost sample preparation and accurate, precise quantification. We utilized a rapid and robust sample preparation method and developed a technique capable of separating ganglioside isomers using a ZIC-HILIC column in a 25 min runtime. To overcome the complexities of ganglioside studies, including variations in extraction efficiency and matrix interference, we employed internal standards—compounds of a similar structure, usually heavily labeled. These internal standards, added at a known concentration, help account for variations in sample preparation, extraction efficiency, and instrumental bias, providing a more accurate and reproducible quantification of gangliosides [20]. Accurate and automated data processing is also crucial for ensuring reliable and fast data output. Ganglioside data processing involves several essential steps—detecting peaks corresponding to ganglioside analytes in the mass spectra, integrating these peaks to measure their area, and quantifying them by comparing the integrated peaks to a calibration curve generated from the internal standard and known concentrations of the gangliosides [20,21].

These optimized methods were applied to study gangliosides in cell lines from pancreatic cancer, breast cancer, and neuroblastoma—some of the most aggressive and deadly cancers in the United States [22]. Gangliosides are of particular interest in these cancers due to their involvement in cell proliferation, differentiation, and migration. High expression of ganglioside GM2 has been reported in pancreatic ductal adenocarcinoma (PDAC) and linked to growth, invasion, and advanced stage [23], while ganglioside GD2 is highly expressed on the surface of neuroblastoma cells and has emerged as a potential immunotherapy target [24,25]. In this study, we developed a fast, robust, and antibody-free analytical methodology for “gangliosidomics”, enabling the separation and quantification of various gangliosides, including isomers, in a single LC run. To the best of our knowledge, this is the first method to use ZIC-HILIC LC-MS for the separation and quantification of multiple gangliosides from biological samples. Additionally, we developed an R script to filter data from Lipid Search output and automate data processing, significantly increasing processing speed. The parameters of the script were based on rigorous manual data validation. Overall, the optimized LC-MS method, combined with fast and robust sample preparation, enabled accurate, precise, and rapid ganglioside analysis in pancreatic cancer, breast cancer, and neuroblastoma cell lines.

## 2. Materials and Methods

### 2.1. Chemicals and Reagents

HPLC-grade water (H_2_O), acetonitrile (ACN), and MS-grade ammonium acetate were obtained from Fisher Scientific (Fair Lawn, NJ, USA). Ammonium bicarbonate (ABC), HPLC-grade methanol, and dichloromethane were purchased from Sigma-Aldrich (St. Louis, MO, USA). Ganglioside standards GD1a, GD1b, GD2, GM1, GT1a were purchased from EMD Millipore Corp. (Billerica, MA, USA), and GM_1_d_3_ (internal standard) was purchased from Cayman Chemical (Ann Arbor, MI, USA).

### 2.2. Stock Solutions of Ganglioside Standards

The stock ganglioside standards (GD1a, GD1b, GD2, GM1, and GT1) and the internal standard (GM_1_d_3_) with a purity of approximately 99% deuterated forms (d_1_–d_3_) were dissolved in 50:50 mixture of H_2_O and ACN and then diluted to a concentration of 1 µg/µL. These standard solutions were stored at −20 °C. Stock samples were further diluted to varying concentrations of 30 ng/µL, 100 ng/µL, 300 ng/µL, 500 ng/µL, 700 ng/µL, and 1000 ng/µL which were retained for use in LC-MS method validation. The internal standard (IS) was diluted to concentrations of 500 ng/µL and 1 µg/µL.

### 2.3. Cell Lysis for Sphingolipid Extraction

The COG-N-683 neuroblastoma cell line was obtained from the Children’s Oncology Group, Alex’s Lemonade Stand Foundation Childhood Cancer Repository (www.CCcells.org). Approximately 20 million cells were counted and collected for sphingolipid profiling. The pancreatic adenocarcinoma cell line, PSN1, was provided by the Haabs group. Cultures of the breast cancer cell line, MDA-MB-231BR, were generously provided by Dr. Paul Lockman (West Virginia University, School of Pharmacy, Morgantown, WV, USA), and the brain cancer cell line, CRL-1620, was purchased from the American Type Culture Collection (ATCC, Manassas, VA, USA). These biological samples (CRL-1620, PSN1, MDA-MB-231BR, and COG-N-683) were used to test the optimized method.

Cells were lysed in a homogenization buffer (50 mM ammonium bicarbonate with a pH of 8.0) using five cycles of bead beating with zirconium beads at 4 °C for 30 s intervals. The lysates were then sonicated on ice for 60 min. After sonication, the lysate was centrifuged at 21,100× *g* for 15 min, and the supernatant was collected. Two µL of the homogenate was used to determine the protein concentration of the cancer cell line samples using the micro-BCA protein assay, following the vendor’s recommended procedure (Thermo Scientific/Pierce, Rockford, IL, USA).

### 2.4. Optimization of Sphingolipids Extraction

Two methods of sphingolipid extraction were tested, and the percent recovery of each method was evaluated. Following this, the number of identified sphingolipids after LC-MS runs was analyzed using LipidSearch 4.2 Software (Thermo Fisher Scientific, Waltham, MA, USA). The first extraction method was Folch extraction [26], where the homogenate was mixed with chloroform and methanol in a 2:1 (*v*/*v*) ratio, along with 0.9% NaCl. The second method used 100% fresh methanol, mixed with the homogenate at a 1:7 volume ratio. Initially, 72 µL of homogenate was combined with 504 µL of absolute methanol, and the mixture was vortexed vigorously for 5 min. However, as part of the optimization process, to enhance the intensity of identified sphingolipids in the PSN1, MDA-MB-231BR, and CRL-1620 cell lines, 70 µL of homogenate was instead mixed with 450 µL of absolute methanol. The mixture was then vortexed and centrifuged at 21,100× *g* for 15 min. The clear supernatant containing sphingolipids was carefully pipetted into a separate vial. For both methods, we compared the number of lipid identifications, percent recovery, and the intensity of extracted sphingolipids.

### 2.5. Optimization of LC Separation Techniques for Analysis of Intact Gangliosides

The separation conditions for sphingolipids were optimized by comparing two columns: the Acclaim^®^ C18 column (3 μm, 300 Å, 150 × 2.1 mm; Thermo Scientific, Sunnyvale, CA, USA) and the ZIC-HILIC column (3.5 μm, 200 Å, 150 × 2.1 mm; Millipore Sigma, Darmstadt, Germany). We evaluated the total ion chromatogram (TIC) generated from each column. Additionally, the ability to resolve the isobars of GD1 was used as a key criterion for selecting the optimal column for further experiments. To confirm the elution order of GD1a and GD1b, we injected mixed concentrations of GD1a and GD1b standards in ratios of 3:1 and 2:3.

### 2.6. Negative Mode LC-MS Setup

The ganglioside standards were analyzed using a Dionex Ultimate 3000 analytical LC system (Sunnyvale, CA, USA) connected to a Q-Exactive HF Orbitrap mass spectrometer. A mixture of five ganglioside standards (GD1a, GD1b, GD2, GD3, GM3) at different concentrations was analyzed at flow rates of 0.1 mL/min and 0.2 mL/min. This mixture was also analyzed using LC-MS. Two solvent systems, mobile phase A (MPA) and mobile phase B (MPB), were combined to form a binary gradient. MPA consisted of 90% acetonitrile, 10% H_2_O, and 5 mM ammonium acetate, while MPB was composed of 100% HPLC water with 5 mM ammonium acetate. The pump operated at an optimized flow rate of 0.2 mL/min with 60% MPB for loading the sample onto the enrichment trap connected to the ZIC-HILIC column. The LC run lasted 25 min, programmed as follows: MPB was gradually increased to 60% over the first 14 min, held constant at 60% for 5 min, and then returned to 0% at 20 min until the end of the run.

Data acquisition was performed using Data Dependent Acquisition (DDA) in negative mode. The mass spectrometer was set to conduct Full MS and all-ion fragmentation (AIF) scan experiments. The FTMS scan range was 500–2000 *m*/*z* with a mass resolution of 120k, while the AIF scan range was 133.3–1999.5 *m*/*z* with a normalized collision energy of 30%, using the high-energy collision dissociation (HCD) technique.

### 2.7. Sphingolipid Profiling in Cog-N-683, MDA-MB-231BR, CRL-1620, and PSN1 Cancer Cell Lines

A database containing the accurate monoisotopic mass with isotopic distribution for ganglioside structures, including varying ceramide moieties (chain length variation) and charge state information (doubly and singly deprotonated ions), was generated. GD1a, GD1b, GD2, GM1, and GD3 standards were run on the LC-MS to determine ganglioside retention times. Chromatograms were extracted using ThermoFisher Xcalibur software 4.1.50 based on *m*/*z* ratios from different ceramide moieties and isotopic distributions for each ganglioside, within a tolerance of 10 ppm. True ganglioside peaks within the mass tolerance were integrated for quantitation. This manual inspection was critical for the later development of automated quantitation parameters.

Building on this manual inspection, the next round of data processing was conducted using LipidSearch 4.2 Software (Thermo Fisher Scientific) for ganglioside identification and quantitation. The raw files from the LC-MS experiment were imported, with parameters set as follows: parent tolerance at 10 ppm, product tolerance at 10 ppm, merge range (min) at 0.1, and absolute intensity threshold at 5000. The top rank filter, all isomer peaks, and FA priority options were activated. The RT range (min) was set to ±0.5. Detection was configured for negative ions, with -H and -2H as the chosen adducts. All sphingolipids were selected as lipids of interest. For validation, the sphingolipids reported by the software were cross-checked with lipid maps and verified manually.

We also developed R scripts to clean and filter the LipidSearch results. Briefly, our criteria for sphingolipid inclusion were a retention time of more than 2 min, a peak area greater than 1000, and a t-score less than 0.5. These parameters were chosen after manually reviewing batches of results to distinguish true analyte signatures from false positives. We believe these criteria provide a sound foundation for data processing. Utilizing these R scripts significantly reduced data processing time. The nomenclature of sphingolipids in LipidSearch 4.2 software is provided in Table S1.

## 3. Results

**Figure 1:**
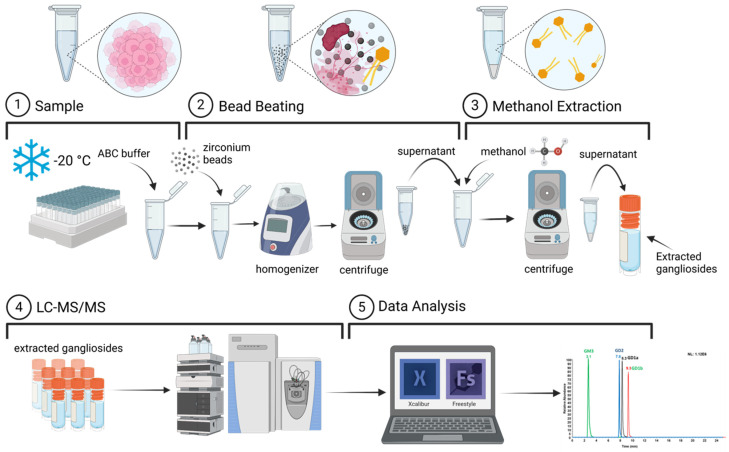
Experimental workflow for ganglioside extraction and analysis, including sample preparation, LC-MS, and data analysis. Cells were lysed using zirconium beads and sonicated on ice for 60 min. The protein concentration of each cell line was determined. Homogenates were then centrifuged, and the supernatant was collected for sphingolipid extraction. The extracted gangliosides were subsequently analyzed using LC-MS with negative mode detection.

Changes in ganglioside levels are linked to various diseases, making them important targets for investigation. However, ganglioside analysis is challenging due to the need for more cost-effective extraction methods and improved accuracy, as their low concentration and the presence of complex biomolecular matrices often interfere with detection and analysis. In this study, a reliable, rapid, and cost-effective method for profiling gangliosides was optimized, with a focus on achieving isomeric separation of intact species. This involved the optimization of an LC-MS method alongside ganglioside extraction techniques. Figure 1 illustrates the protocol followed for the optimized ganglioside analysis, covering both sample preparation and data processing steps. Briefly, cells were lysed in a 50 mM ABC buffer via bead beating with zirconium beads.

Following the bead beating, the lysates were sonicated on ice, followed by centrifugation, and the supernatants from each were collected. The protein concentration of each cell line was determined using a micro-BCA protein assay. Sphingolipid extraction was carried out followed by LC-MS analysis in the negative ion mode. The raw data were processed using LipidSearch 4.2 and Xcalibur 4.1.50 softwares. The aim of this investigation is to optimize sphingolipid extraction and the LC-MS method. To optimize the extraction protocol, we compared the percentage recovery between the Folch extraction method and the use of absolute methanol. Figure S1 presents the calibration plot of the internal standard used to evaluate the percentage recovery for both extraction methods. Based on three biological replicates, the absolute methanol extraction method yielded a higher percentage recovery, as shown in Table 1. The first replicate in the MeOH extraction method yielded more than 100%; a possible reason is that the internal standard reagent is not 100% pure (it has a percent purity of ≥99%), which might have slightly interfered with the percent recovery.

**Figure 2:**
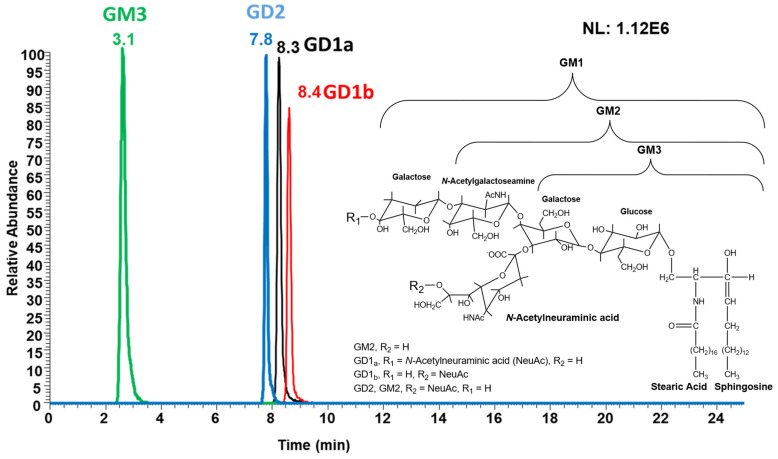
EIC traces for GM3, GD2, GD1a, and GD1b, including their structural derivatives. The structures of each ganglioside are shown in the inset, with the designations of each abbreviated functional group provided as a legend within the inset.

The extracted ion chromatogram (EIC) of ganglioside standard GM3, GD2, GD1a, and GD1_b_ obtained from the LC-MS method is shown in Figure 2, along with their structures. GM3 eluted earlier compared to other ganglioside standards, likely due to its single sialic acid, prompting limited interaction with the column materials.

### 3.1. Optimization of LC Separation

The separation of analytes, especially isomers, is crucial for accurate quantitation and identification in LC-MS analysis. Gangliosides have several biologically relevant isomers, making precise separation essential. Various flow rates were tested using a ZIC-HILIC column to optimize the separation of gangliosides and their isomers. Optimal separation of GD1a and GD1b isomers was achieved by adjusting the flow rate from 0.1 mL/min to 0.2 mL/min.

**Figure 3:**
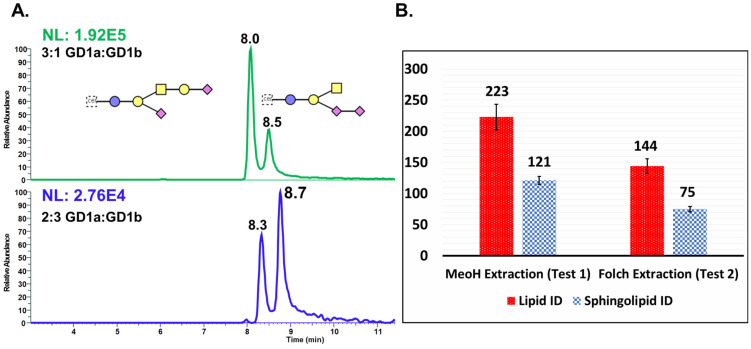
The (**A**) 3:1 and (**B**) 2:3 mixtures of GD1a and GD1b standards with isomeric chromatographic separation. This provides verification for the or der of elution of GD1a and GD1b. (**B**) Side-by-side comparison of Folch and absolute methanol extraction methods. Monosaccharide symbol legends: glucose (blue circle), galactose (yellow circle), *N*-acetylgalactosamine (yellow square), and *N*-acetylneuraminic acid (magenta diamond).

**Figure 4:**
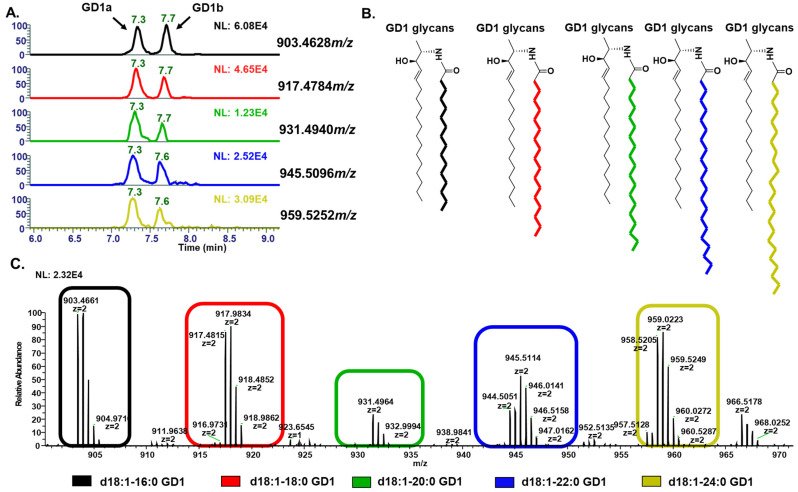
(**A**) Extracted ion chromatogram for GD1a and GD1b isomers in biological sample at different *m*/*z*, (**B**) because of varying ceramide moiety in each GD1 structures, which shows the complexity of gangliosides. (**C**) MS traces corresponding to each GD1 ion chromatogram.

Figure 3A presents the stacked EICs for 3:1 and 2:3 mixtures of GD1a and GD1b, which were used to confirm the elution order of these isomers. Figure 3B shows a side-by-side comparison of the absolute methanol and Folch extraction methods. The methanol extraction method yielded a higher number of sphingolipids, with an average of 121 sphingolipid IDs, compared to 75 IDs with the Folch method. The absolute methanol extraction produced an average of 223 lipid IDs, whereas the Folch method yielded 144 lipid IDs. Figure 4A shows the EIC of GD1a and GD1b in a biological sample across varying *m*/*z* values of GD1, and the differences in the fatty acyl chain length is shown in Figure 4B. The MS trace of each ion chromatogram corresponding to each *m*/*z* of GD1 is shown in Figure 4C. This highlights the complexity of gangliosides due to variation in the fatty acid chain length. Calibration plots for GM3, GD1, GD2, and GD3 were generated, with R-squared values ranging from 0.9961 to 0.9975. The analytical merits of the optimized method, including the limits of detection (LOD) and quantitation (LOQ), are detailed in Table S2. The extracted ion chromatogram (EIC) for *m*/*z* 931.4940 (M-2H)^2−^, d18:1-20:0, one of the primary masses for GD1, in the two LC separation methods is shown in Figure S2.

Compared to 0.1 mL/min, with 0.2 mL/min, the elution time was reduced from almost 16 min (in 0.1 mL/min) to around 8 min (in 0.2 mL/min). This can lend itself to shorter LC gradients, therefore reducing analysis time. One interesting facet was the multiple peaks that were revealed by the faster flow gradient (Figure S2). We concluded these were still GD1 isomers, and explanations include differing lipid chain configurations such as cis vs. trans double bonds, which can dramatically affect the three-dimensional space of the ganglioside, in turn affecting column–analyte interaction.

### 3.2. Ganglioside Profiles of Cog-N-683, MDA-MB-231BR, CRL-1620, and PSN1

**Figure 5:**
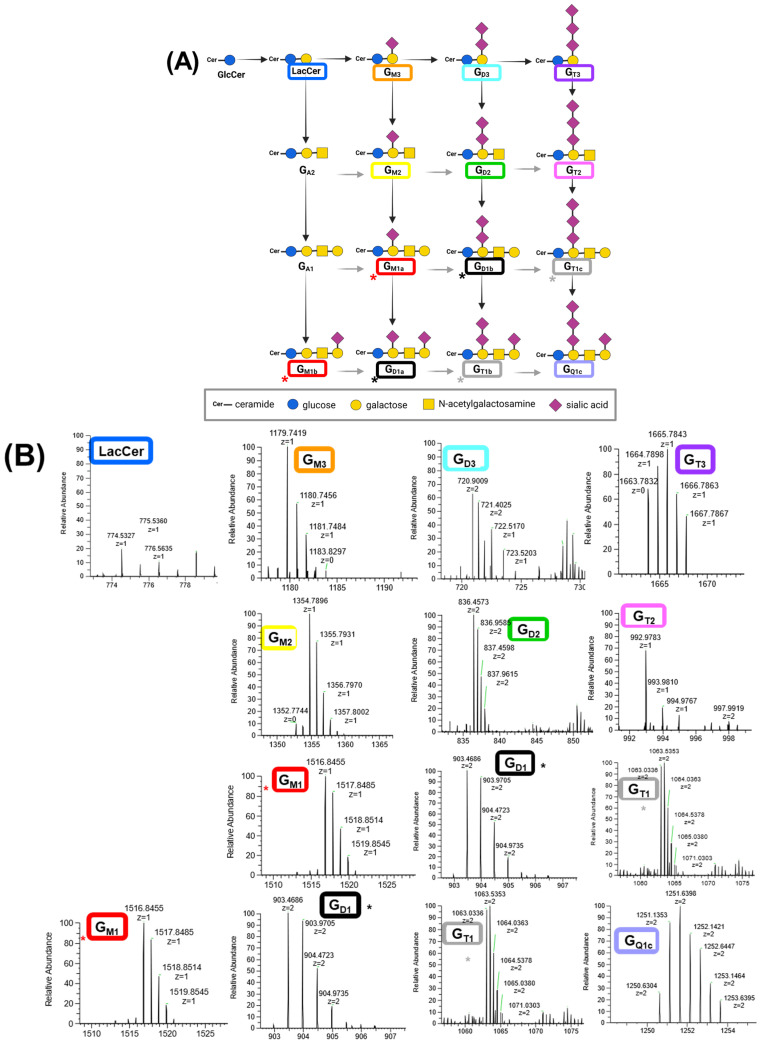
(**A**) Ganglioside biosynthesis pathway and (**B**) their MS traces in biological samples. Isomers are indicated by an asterisk (*) next to the name. The MS trace corresponding to each ganglioside in the pathway are highlighted in a similar colored textbox.

**Figure 6:**
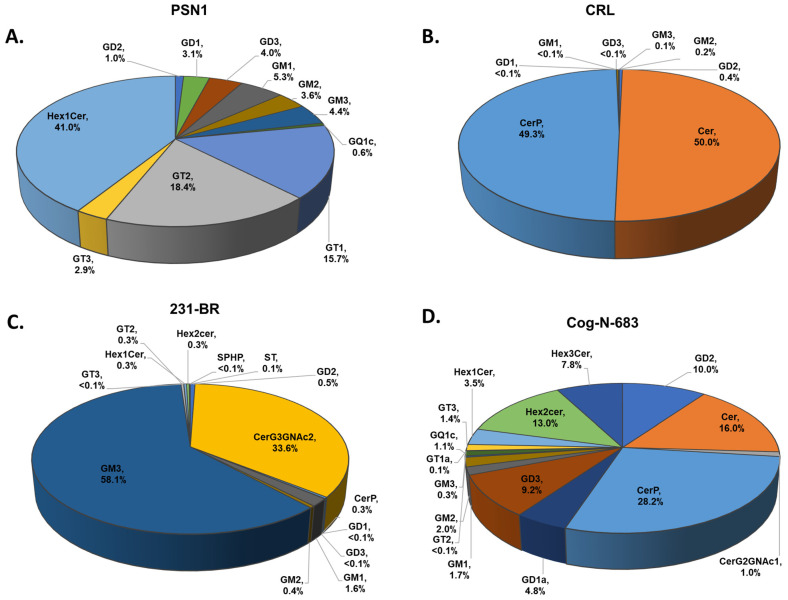
Pie charts of the average relative abundance of gangliosides in (**A**) PSN1, (**B**) CRL-1620, (**C**) MDA-MB-231BR, and (**D**) COG-N-683.

Using the developed method for ganglioside extraction and profiling, we analyzed four cell lines: PSN1, CRL-1620, 231 BR, and COG-N-683. The ganglioside biosynthesis pathway [27] and MS traces of the gangliosides identified in the cell lines are shown in Figure 5. Notably, ganglioside GA_1_ and GA_2_ were not detected in the cells. The *m*/*z* and charge states are highlighted in a colored textbox, which corresponds to the color used to highlight each ganglioside in the pathway. Table 2 includes the *m*/*z* values of the precursor ions (both theoretical and observed) for each ganglioside detected in the biosynthesis pathway, along with the adducts used to extract the MS traces and the mass accuracy of each detected ganglioside. The resulting ganglioside profiles for each cell line are shown in Figure 6A–D, with a brief description of each cell line provided in Table 3. Eleven sphingolipids were identified and quantified in PSN1, as illustrated in Figure 6A.

Briefly, Hex1Cer was the most abundant sphingolipid in PSN1 with a percentage relative abundance of 41.00 ± 0.08, followed by GT2 with 18.40 ± 0.05 and then GT1, GM1, GM3, GD3, GM2, and GD1 with a percentage relative abundance of 15.70 ± 0.01, 5.30 ± 0.01, 4.400 ± 0.001, 4.00 ± 0.01, 3.60 ± 0.01, and 3.100 ± 0.001, respectively.

In CRL-1620, eight sphingolipids were identified and quantified, and Ceramide (Cer) and Ceramide phosphates (CerP) were the most abundant, as shown in Figure 6B, with a percentage relative abundance of 50.0 ± 0.5 and 49.3 ± 0.8, respectively. Fourteen sphingolipids were identified and quantified in MDA-MB-231BR. As shown in Figure 6C, in this cell line, GM3 was the most abundant sphingolipid with a percentage relative abundance level of 58.1 ± 0.9, followed by CerG_3_GNAc_2_, which was 33.6 ± 0.8, and then GD2, GM1, and GM2 with a percentage relative abundance level of 4.90 ± 0.01, 1.60 ± 0.09, and 0.40 ± 0.07, respectively. Sulfatide (ST) and sphingosine phosphate were also identified and quantified in 231-BR, with a percentage relative abundance of 0.10 ± 0.01 and < 0.10 ± 0.07, respectively.

**Figure 7:**
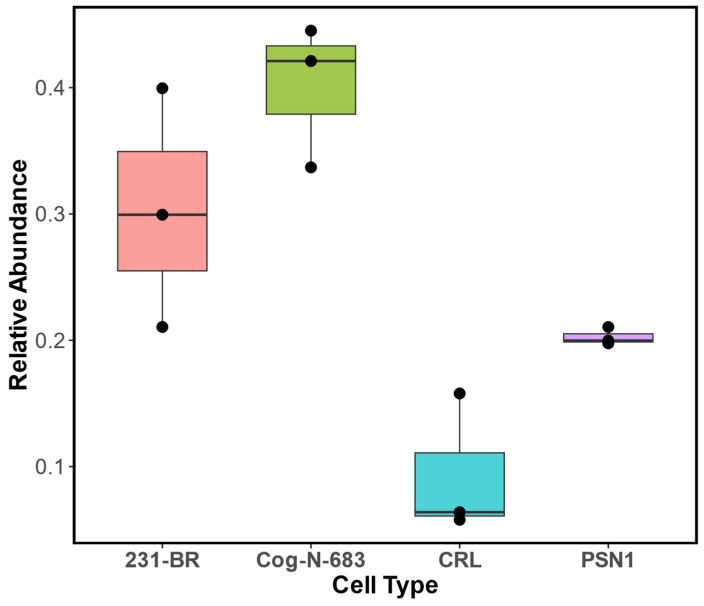
Boxplot of the relative abundance of all sphingolipids identified and quantified in cell lines from metastatic breast cancer (MDA-MB-231BR), neuroblastoma (COG-N-683), brain cancer cell line (CRL-1620), and pancreatic cancer cell line (PSN1). The total relative abundance for sphingolipids in each cell line was summed for each replicate (*n* = 3).

In Figure 6D, the sphingolipid profile for COG-N-683 is shown. Sixteen sphingolipids were identified and quantified. CerP, Cer, Hex2cer, GD2, GD3, Hex3Cer, and GD1 were the most abundant, and their percentage relative abundance levels were 28.20 ± 0.91, 16.0 ± 0.9, 13.0 ± 0.6, 10.0 ± 0.2, 9.2 ± 0.2, 7.80 ± 0.05, and 4.8 ± 0.8, respectively. Overall, the neuroblastoma cell line COG-N-683 had the highest number of sphingolipid species (16 different sphingolipids), followed by MDA-MB-231BR (14 different sphingolipids), PSN1 (11 different sphingolipids), and CRL-1620 (8 different sphingolipids). In addition, COG-N-683 had the highest relative abundance of sphingolipids (0.40 ± 0.04), followed by PSN1 (0.200 ± 0.005), MDA-MB-231BR (0.40 ± 0.05), and CRL-1620 (0.09 ± 0.05), respectively. A bar plot comparing the relative abundance of sphingolipids in the four cell lines is shown in Figure 7.

## 4. Discussion

We have developed and optimized an LC-MS method for profiling sphingolipids, specifically gangliosides, across various biological samples. This method was crucial due to the complex and heterogeneous structures of gangliosides. These molecules are involved in key physiological processes such as cell growth, viral transformation, and tumor progression, making them potential targets for cancer immunotherapy and molecular characterization [34]. This method demonstrated the effective separation and quantitation of ganglioside isomers. Ganglioside analysis is particularly challenging due to the varying *m*/*z* values for the same ganglioside analyte, which can differ based on fatty acid chain length or glycan head groups. Therefore, separating ganglioside isomers requires a selective and efficient column like ZIC-HILIC, as well as a high-resolution mass spectrometer. In a study that compared separation of glucosylceramide on a ZIC-HILIC column to an unmodified silica gel column, ZIC-HILIC was reported to be a valuable tool for characterizing the structural diversity of mono-glucosylated lipids in biological material and for quantifying these important lipids [35]. Additionally, we achieved a shorter sample preparation process for ganglioside profiling without the need for antibody tagging.

An innovative yet instrumentally complex study utilized an ion polarity switching LC-MS approach to investigate the ganglioside isomers in mouse brain samples [36]. While Li et al. were able to identify 165 gangliosides and 100 isomers using this method, this requires not only multiple instruments (GC-MS and LC-MS) but also advanced instrumental expertise to couple mass spectrometry instruments together [36]. In-depth ganglioside isomer characterization is useful in many applications; however, it does require additional instrumentation, analysis time, and sample preparation compared to our method. Our method requires only one LC-MS system with no physical modifications being made.

Characterizing gangliosides using LC-MS is advantageous due to the high throughput and sensitivity of this analytical tool. However, not all columns can efficiently separate gangliosides and other lipid species in a single run. Lee et al. used a C18 column for the separation of cell membrane gangliosides in cancer cells. This method was used to investigate cell surface gangliosides; however, it was unable to separate ganglioside isomers [37]. This is most likely due to the non-polar material used in the column. With a ZIC-HILIC column, the interactions of the analyte with the column phase favors separation of gangliosides by their head group rather than the lipid tail. This leads to an increased ability to separate isomers such as GD1a and GD1b. Zhang et al. developed a method to investigate ganglioside–protein interactome using bifunctional probes, but only a few gangliosides could be targeted [38]. The development of high-throughput methods such as ours may provide needed advancements for the adoption of these types of techniques.

Liquid–liquid partitioning remains the most common method for ganglioside extraction from various sample sources [39]. Sphingolipid extraction from cell lines using absolute methanol resulted in higher sphingolipid identification, percent recovery, and intensity compared to the conventional Folch extraction method. The use of absolute methanol for lipid extraction has been successfully applied in protocols for various sample types, including serum, plant tissues, and animal tissues [40,41]. In this study, we optimized the extraction method to obtain gangliosides and other sphingolipids from cell lines more effectively. A successful solvent extraction method depends on several key factors, including its selectivity and stability for the lipid type of interest, as well as its ability to minimize artifacts [42]. Since our focus was on sphingolipids, we further evaluated the percentage of sphingolipids identified by the two extraction methods. Absolute methanol yielded the highest percentage recovery, at 96 ± 7%, while the Folch method achieved a recovery of 91 ± 8%. These results are consistent with findings previously reported by Eggers et al., 2016, where the percent recovery for the Folch method was also above 90% [43].

Optimization of the LC-MS analysis involved examining several factors, including the choice of LC column for ganglioside separation, the pump flow rate, and the experiment’s run-time. The efficiency of the method was evaluated based on its ability to resolve GD1 isomers (GD1a and GD1b), which served as a key analytical metric. In a previous study reported by Schindler et al., 2024 [44], they were unable to separate GD1a and GD1b isomers, likely due to limitations in the chromatography method. In our study, we tested pump flow rates of 0.1 mL/min and 0.2 mL/min with experimental run times of 25 and 30 min. The optimal conditions were determined to be a flow rate of 0.2 mL/min and a 25 min run time. The ZIC-HILIC column, which provided superior resolution for separating GD1 isomers, has previously been reported to be highly sensitive and to offer excellent resolution for analyte separation across various “omics” fields [45,46,47,48,49].

The optimized ganglioside profiling method provided valuable insights into the distribution of gangliosides in neuroblastoma, breast cancer, and pancreatic cancer cell lines, while maintaining high levels of accuracy and sensitivity. Additionally, our findings confirmed that ganglioside diversity is more influenced by the glycan head group than by the ceramide moiety, as previously reported [37].

In this study, we identified potential biomarkers previously reported for cancerous cell lines, including pancreatic and breast cancer, as well as serum, specifically GM3, GM1, and GM2. Among these, GM3 was found to be the most abundant in the breast cancer cell line (MDA-MB-231BR), while GM1 was highly abundant in the pancreatic cancer cell line (PSN1) compared to other cell lines. These findings align with previous studies that reported high monosialylated gangliosides in pancreatic cell lines [37,50]. GD2 has been a target for neuroblastoma cell lines, and most studies on neuroblastoma focus on GD2 expression and quantification, usually by flow cytometry [51,52]. Studies have also shown that the GD2 expression level is high in breast cancer [53,54,55], and, interestingly, GD2 is among the top five most abundant sphingolipids identified in this study. However, the sensitivity, high resolution separation, and ability of the LC-MS technique to detect and quantify a wide range of different types of gangliosides in a single run makes it the gold standard. ST and SPHP phosphates (SPHP) were also identified and quantified in MDA-MD-231BR. Previous studies have suggested that STs are abundantly expressed in certain cancers and tumors, while SPHP plays a significant role in inflammation and cancer progression [56,57].

## 5. Conclusions

In this study, we optimized extraction conditions, LC-MS analysis, and data processing to enable easier, faster, and more reliable analysis of gangliosides and other sphingolipids. To the best of our knowledge, this is the first method to use the ZIC-HILIC column on LC-MS to separate and quantify multiple gangliosides from biological samples. Future studies could expand the analysis to a broader variety of cancer cell lines and serum samples, with a larger number of biological replicates, to identify additional ganglioside and sphingolipid biomarkers. Such research can potentially translate into clinical applications for diagnosis and therapeutic development.

## Figures and Tables

**Table 1:** Efficiency of two sphingolipid extraction protocols with three biological replicates.

Extraction Methods	1st Replicate (%)	2nd Replicate (%)	3rd Replicate (%)	Average
Folch extraction	98.2	94.3	81.5	91.4 ± 8
MeOH extraction	103.4	96.4	89.2	96.3 ± 7

**Table 2:** Gangliosides in ganglioside biosynthesis pathways detected in cell lines by using LC-MS.

Ganglioside Types	Adduct	Theoretical *m/z*	Observed *m/z*	Mass Accuracy (ppm)
GlcCer	Hex1Cer(d17:0_18:2+2O)-H	742.5475	742.5424	6.8
LacCer	Hex2Cer(m17:1_12:0)-H	774.5373	774.5327	6.1
GM3	GM3(d18:1_18:0)-H	1179.7365	1179.7419	4.5
GD3	GD3(d18:1_16:0)-H	720.8967	720.9009	4.5
GT3	GT3(t16:1_12:0)-H	1664.7982	1664.7898	5.0
GD2	GD2 (d18:1_18:0)-2H	836.4520	836.4574	6.4
GT2	GT2(d18:1_21:5)-2H	992.9761	992.9785	2.4
GM1	GM1(d18:1_16:0)-H	1516.8375	1516.8451	5.0
GT1	GT1(d18:1_18:0)-2H	1063.0261	1063.0338	7.3
GD1	GD1 (d18:1_18:0)-2H	903.4628	903.4687	6.5
GQ1	GQ1(d18:1_18:0)-2H	1250.6206	1250.6333	10.0
GM2	GM 2 (d18:1_16:0)-H	1354.7847	1354.7892	3.6

**Table 3:** Relative abundance of sphingolipids across various cancer cell lines with a brief description of each cell line type.

Cell Line	Description	Sphingolipids	Abbreviation	%Relative Abundance
CRL-1620	Established from tumor tissue of a glioblastoma patient [28].	Ceramide	Cer	50.0 ± 0.5
		Ceramides phosphate	CerP	49.3 ± 0.8
		Disialoganglioside-GD2	GD2	0.37 ± 0.08
		Monosialoganglioside-GM2	GM2	0.20 ± 0.05
		Monosialoganglioside-GM3	GM3	0.1 ± 0.2
		Monosialoganglioside-GM1	GM1	<0.10 ± 0.01
		Disialoganglioside-GD3	GD3	<0.10 ± 0.01
		Disialoganglioside-GD1	GD1	<0.10 ± 0.01
PSN1	Established from a pancreatic adenocarcinoma [29,30].	Hexosylceramide	Hex1Cer	41.00 ± 0.08
		Trisialoganglioside-GT2	GT2	18.40 ± 0.05
		Trisialoganglioside-GT1	GT1	15.70 ± 0.01
		Monosialoganglioside-GM1	GM1	5.30 ± 0.01
		Monosialoganglioside-GM3	GM3	4.400 ± 0.001
		Disialoganglioside-GD3	GD3	4.00 ± 0.01
		Monosialoganglioside-GM2	GM2	3.60 ± 0.01
		Disialoganglioside-GD1	GD1	3.100 ± 0.001
		Trisialoganglioside-GT3	GT3	2.90 ± 0.01
		Disialoganglioside-GD2	GD2	1.00 ± 0.01
		Tetrasialoganglioside-GQ1c	GQ1c	0.60 ± 0.01
231-BR	MDA-MD-231BR established from breast cancer [31].	Monosialoganglioside-GM3	GM3	58.1 ± 0.9
		Simple Glc series	CerG3GNAc2	33.6 ± 0.8
		Disialoganglioside-GD2	GD2	4.90 ± 0.01
		Monosialoganglioside-GM1	GM1	1.60 ± 0.09
		Monosialoganglioside-GM2	GM2	0.40 ± 0.07
		Trisialoganglioside-GT2	GT2	0.30 ± 0.01
		Hexosylceramide	Hex2Cer	0.30 ± 0.02
		Hexosylceramide	Hex3Cer	0.30 ± 0.01
		Ceramides phosphate	CerP	0.30 ± 0.05
		Sulfatide	ST	0.10 ± 0.01
		Disialoganglioside-GD1	GD1	<0.10 ± 0.01
		Trisialoganglioside-GT3	GT3	<0.10 ± 0.05
		Sphingosine-1-phosphate	SPHP	<0.10 ± 0.07
		Disialoganglioside-GD3	GD3	<0.10 ± 0.08
COG-N-683	Established at time of diagnosis from a neuroblastoma bone marrow metastasis [32,33].	Ceramides phosphate	CerP	28.2 ± 0.5
		Ceramide	Cer	16.0 ± 0.9
		Hexosylceramide	Hex2Cer	13.0 ± 0.6
		Disialoganglioside-GD2	GD2	10.0 ± 0.2
		Disialoganglioside-GD3	GD3	9.2 ± 0.2
		Hexosylceramide	Hex3Cer	7.80 ± 0.05
		Disialoganglioside-GD1	GD1	4.8 ± 0.8
		Hexosylceramide	Hex1Cer	3.5 ± 0.8
		Monosialoganglioside-GM2	GM2	2.00 ± 0.09
		Monosialoganglioside-GM1	GM1	1.70 ± 0.08
		Trisialoganglioside-GT3	GT3	1.40 ± 0.07
		Tetrasialoganglioside-GQ1c	GQ1c	1.10 ± 0.03
		Simple Glc series	CerG2GNAc1	1.00 ± 0.09
		Monosialoganglioside-GM3	GM3	0.30 ± 0.09

## Data Availability

The raw files collected from this study have been submitted to GlycoPOST [58] and can be accessed at the following link: https://glycopost.glycosmos.org/preview/1269600027667984f46c0b2 Pin Code: 6650. The script used to process LipidSearch results can be found on GitHub at https://github.com/andybennett7/ganglioside_method_paper.git.

## Supplementary Materials

The following supporting information can be downloaded at https://www.mdpi.com/article/10.3390/cells13191640/s1, Table S1: Nomenclature of sphingolipids in LipidSearch 4.2 software; Table S2: Analytical merit obtained from calibration plots of standard ganglioside analysis; Figure S1: Calibration plot of internal standard (GM1_d3) used for evaluating percentage recovery of both Folch and absolute methanol extraction methods; Figure S2: Extracted ion chromatogram (EIC) and MS traces for GD1 isomers (*m*/*z* 931.4940). (A) ZIC-HILIC column with 0.1 mL/min. (B) ZIC-HILC column with 0.2 mL/min.
