# Supplementary material for: An Optimized Liquid Chromatography–Mass Spectrometry Method for Ganglioside Analysis in Cell Lines
Source: Cells. 2024 Oct 2;13(19):1640. doi: 10.3390/cells13191640 (PMC11476222; doi:10.3390/cells13191640)
Supplement: Supplementary file 1 [file cells-13-01640-s001.zip › cells-3227322-supplementary.pdf]

SUPPORTING INFORMATION

An Optimized LC-MS Method for Ganglioside Analysis in Cell Lines

Akeem Sanni [1‡], Andrew I. Bennett [1‡], Yifan Huang [1], Isabella Gidi [1], Moyinoluwa Adeniyi [1], Judith Nwaiwu [1], Min H. Kang [2], Michelle E. Keyel [2], ChongFeng Gao [3], C. Patrick Reynolds [2], Haab Brian [3], Yehia Mechref [1*]

[1]Chemistry and Biochemistry Department, Texas Tech University, Lubbock, Texas, USA

[2] Cancer Center, School of Medicine, Texas Tech University Health Sciences Center, Lubbock, Texas, USA (TTUHSC)

[3]Van Andel Institute, Grand Rapids, MI

‡ Authors contributed equally to this work

*Correspondence: Department of Chemistry and Biochemistry,

Texas Tech University

Lubbock, TX 79409-1061, USA.

Email: yehia.mechref@ttu.edu

Tel: 806-834-8246

Table S1. Nomenclature of Sphingolipids in LipidSearch 4.2 software

Class	Lipid name
Cer	Ceramides
CerP	Ceramides phosphate
CerPE	Ceramides phosphoethanolamines
Hex1SPH	Glucosylsphingosine
Hex1Cer	Simple Glc series
Hex2Cer	Simple Glc series
Hex3Cer	Simple Glc series
CerG2GNAc1	Simple Glc series
CerG3GNAc1	Simple Glc series
CerG3GNAc2	Simple Glc series
GM3	Gangliosides
GM2	Gangliosides
GM1	Gangliosides
GD1a	Gangliosides
GD1b	Gangliosides
GD2	Gangliosides
GD3	Gangliosides
GT1a	Gangliosides
GT1b	Gangliosides
GT1c	Gangliosides
GT2	Gangliosides
GT3	Gangliosides
GQ1c	Gangliosides

Class	Lipid name
GQ1b	Gangliosides
LSM	Lysosphingomyelin
phSM	Sphingomyelin (phytosphingosine)
SM	Sphingomyelin
SPH	Sphingosine
SPHP	Sphingosine phosphate
ST	Sulfatide
AcHexSiE	AcylGlcSitosterol ester
AcHexStE	AcylGlcStigmasterol ester
AcHexZyE	AcylGlcZymosterol ester
AcHexCmE	AcylGlcCampesterol ester
AcHexChE	AcylGlcCholesterol ester
ChE	Cholesterol ester
D7ChE	Deuterated Cholesterol ester
CmE	Campesterol ester
DG	Diglyceride
D5DG	Deuterated Diglyceride
MG	Monoglyceride
SiE	Sitosterol ester
StE	Stigmasterol ester
TG	Triglyceride
D5TG	Deuterated triglyceride

Table S2. Analytical merit obtained from calibration plots of standard ganglioside analysis.

Ganglioside	LOD (pg)	LOQ (pg)	Trendline equation	R^2 value
GM3	38	116	Y= 344.81x-5182.4	0.997
GD1	47	144	Y= 151.49x-3188.6	0.996
GD2	39	121	Y= 30.796x-713.34	0.997
GD3	38	115	Y= 498.61x-7829.9	0.997

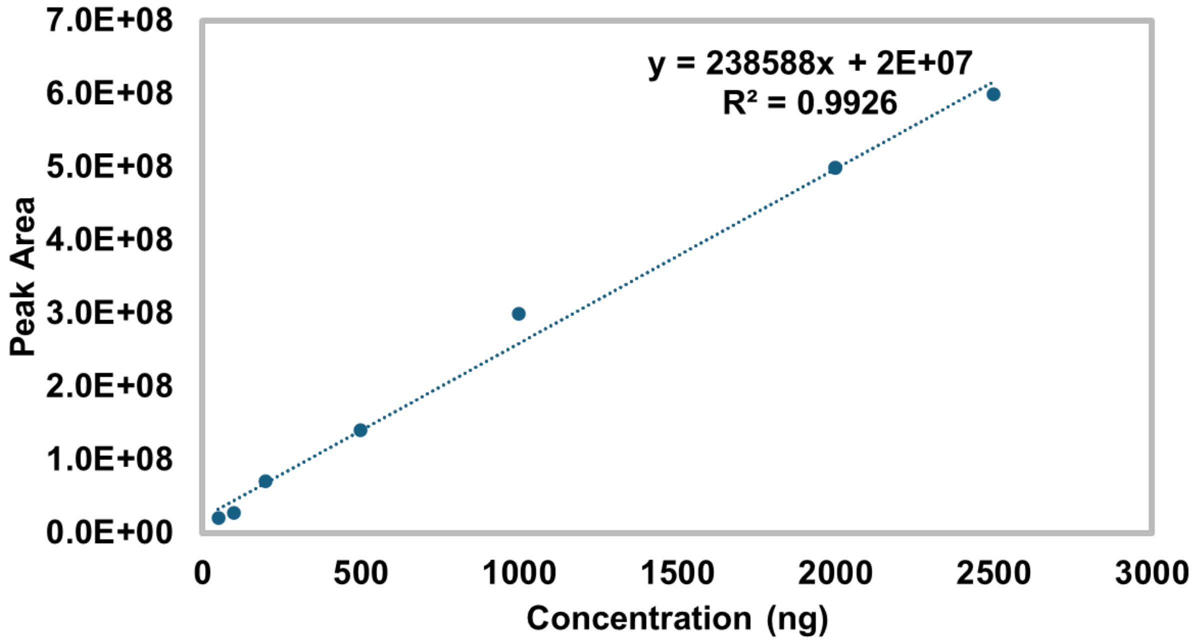

Figure S1. Calibration plot of internal standard (GM1_d3) used for evaluating percentage recovery of both Folch and absolute methanol extraction methods.

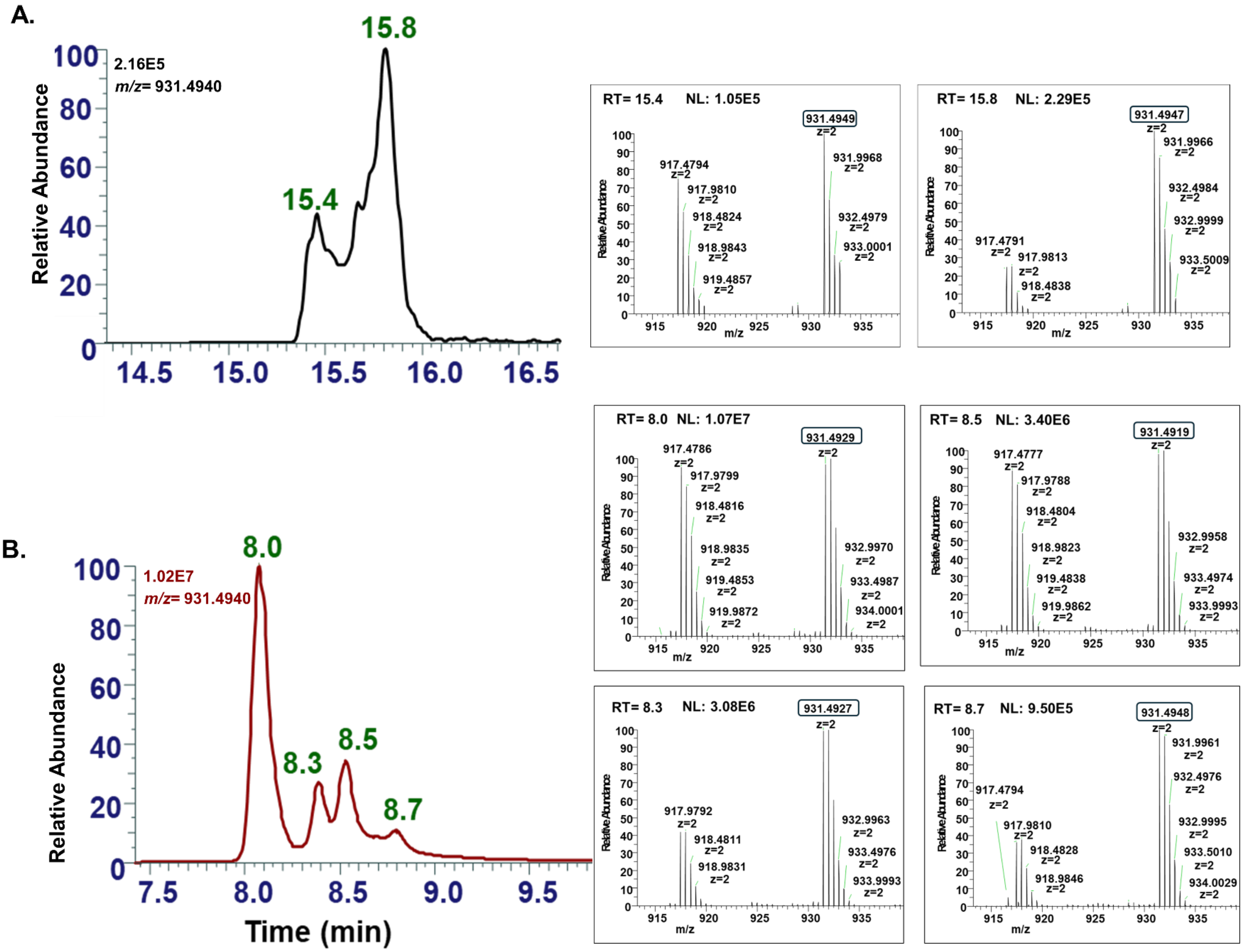

Figure S2. EIC traces for GD1 isomers (*m/z* 931.4940). A) ZIC-HILIC column with 0.1mL/min. B) ZIC-HILIC column with 0.2mL/min.
